# A feasibility and acceptability of virtual reality as a pain relief measure post primary and revision total knee replacement surgery in a hospital setting: quasi-experimental study

**DOI:** 10.1186/s12891-026-09599-y

**Published:** 2026-03-10

**Authors:** Queen Adebola Adeyanju, Boyne Bellew, Maria Joao Cardoso Teixeira

**Affiliations:** 1 Nursing Research Department, Royal National Orthopaedic Hospital NHS Trust, London, UK; 2 NIHR Nursing and Midwifery, London, UK; 3 London South Bank University, London, UK; 4 Nuffield Health, The Manor Hospital, Oxford, UK

**Keywords:** Feasibility, Knee replacement, Non-pharmacological pain management, Virtual Reality

## Abstract

**Background:**

Effective post-operative pain management remains a significant global challenge, especially following joint replacement surgeries. Despite the increasing interest in non-pharmacological interventions, persistent post-surgical pain, opioid dependence, and patient recovery demands continue to drive the search for safer and more cost-effective alternatives. Virtual Reality (VR) has emerged as promising, non-invasive, and drug-free intervention, with increasing evidence supporting its use in managing acute and procedure-related pain. However, its application in patients undergoing total knee replacement (TKR) or revision total knee replacement (RTKR) has not yet been evaluated.

**Methods:**

A single-centre, quasi-experimental feasibility study was conducted over 12 weeks in a UK specialist orthopaedic hospital. Adults undergoing TKR or RTKR were recruited and consented pre-operatively. Beginning 24 h post-surgery, participants were offered up to three VR sessions using the DR.VR system. Each session lasted approximately 7.5 min and included immersive, patient-selected experiences. Pain and anxiety levels were assessed pre- and post-session using validated scales. Acceptability was measured via participant questionnaires following each session.

**Results:**

Of 145 eligible patients, 57 were screened, 42 received study information, and 23 consented to participate: 56.5% female; 43.48%males; mean 69.23, ± 11.64 years. Three withdrew, and 20 participants completed at least one VR session with a retention rate of 87%. In total, 44 sessions were completed. Pain reduction was reported by 81.4% and anxiety reduction by 77.3% of participants post-session. Acceptability was high: 95% enjoyed the experience, 85% felt distracted from pain, and 90% would recommend VR for post-surgical use. No adverse effects were reported. Participant feedback suggested longer session durations and higher-quality content could further enhance the intervention.

**Conclusion:**

This study demonstrates that VR for managing post-operative pain and anxiety in TKR and RTKR patients is a feasible and acceptable non-pharmacological strategy. Findings support its potential as a safe, user-friendly adjunct to standard care. However, further large-scale, multi-centre trials are needed to evaluate efficacy, optimise protocols, and support routine implementation in post-operative settings.

**Trial registration:**

Ethical approval was obtained from Health Research Authority (HRA) and Health and Care Research Wales (HCRW), UK on 27 January 2022 (21/NW/0359).

**Supplementary Information:**

The online version contains supplementary material available at 10.1186/s12891-026-09599-y.

## Introduction

Post-operative pain following joint replacement surgery remains one of the main symptoms and a key predictor of poor clinical outcomes [1]. With the increasing need for improved acute pain management, innovative approaches are being explored. Virtual reality (VR) has emerged as a promising, safe alternative to relieve post-surgical pain [2, 3]. Annually, over 200,000 people in the United Kingdom (UK) undergo joint replacement surgery, accounting for more than 25% of all surgical interventions conducted by the National Health Service [4]. In 2018, around 109,540 knee replacements were performed in the UK [5] and it is projected that by 2060, there would be an estimated 36.6% increase in the number of primary TKRs compared with 2018 [6, 7]. These forecasts, informed by demographic shifts such as the sedentary lifestyle, growing prevalence of obesity, increased life expectancy [3] and an ageing population [8, 9]. Moreover, the high prevalence of osteoarthritis, and earlier diagnoses for patients younger than 55 years [9–11] predicts a significant exponential rise in primary knee replacements through 2030, accompanied by a proportional rise in revision knee replacements [9].

Though pain is one of the main symptoms after any surgery, effective pain management in healthcare remains a major global challenge [12]. Despite the increasing interest in post-operative pain management and development of pain control modalities, often requiring pharmacological interventions, more than half of patients report moderate or severe post-surgical orthopaedic pain [13, 14]. This persistent burden of acute post-operative pain, despite advances in perioperative care, highlights the need to optimise multimodal pain management strategies, including consideration of non-pharmacological adjuncts. Though prescribed opioids are sometimes required, these drugs can negatively affect people’s well-being, social, personal and occupational functioning. Opioids remain an important component of acute post-operative pain management. However, their use is associated with adverse effects and, in some cases, continued use beyond the immediate post-operative period [15]. Concerns about side effects, prolonged use and healthcare costs have prompted interest in non-pharmacological strategies to complement pharmacological care [15, 16]. These findings highlight the need to explore adjunctive approaches to pain management for patients undergoing primary and revision total knee replacement.

VR as an emerging non-pharmacological treatment strategy for pain management has an increasing body of evidence substantiating its positive impact in procedure related pain [17], in hospitalised patients [18, 19], and in chronic and acute lower back pain in both adults and paediatrics [2, 20]. Systematic reviews highlight VR as a promising, effective, non-invasive approach to pain management that needs further research [2, 21]. Importantly, VR is not a standalone intervention, but a concurrent intervention through which a variety of approaches can be delivered, including distraction-based gameplay, immersive nature, travel experiences, mindfulness and relaxation-based exercises. Currently, there is no evidence reporting the use of VR in patients undergoing joint replacement surgery. However, studies in other acute and chronic pain populations suggest that VR-delivered intervention may reduce pain and improve patient experience [22–25]. Therefore, this article reports the feasibility and acceptability of using of VR within a specialist orthopaedic hospital that sets standards for musculoskeletal treatment and provides highly specialised care for patients [26]. In this feasibility study, we did not assess opioid consumption or long-term outcomes. Instead, we focused on the short-term impact of VR sessions on patient-reported pain and anxiety in the acute post-operative period.

## Methods

### Study design

A single-centre quasi-experimental feasibility study to explore the use of VR as an adjunct to standard post-operative pain management for adult patients undergoing knee replacement surgery [27]. The primary objectives was to assess in terms of recruitment and retention. Secondary objectives were to (i) explore changes in patient-reported pain and anxiety levels immediately before and after VR sessions, (ii) assess the acceptability and usability of the VR intervention from the perspective of patient participants, and (iii) explore the feasibility of involving staff and/or volunteers in supporting VR delivery. This project aligns with phase II of the Medical Research Council (MRC) framework for developing and evaluating complex interventions [28, 29], which supports preliminary testing of feasibility and acceptability before larger-scale evaluations [30, 31]. This non-randomised feasibility study is reported in accordance with the TREND statement for non-randomised evaluations of interventions [32] (Additional file 1).

### Study population and recruitment

Study inclusion criteria included age ≥ 18 years; undergoing a primary total knee replacement (TKR) or revision total knee replacement (RTKR); and able to provide written informed consent. Potential participants were identified from elective surgery lists and were approached, study PIS was sent with their pre-operative letter before surgery by the first author or clinical team. At this pre-operative, the study was introduced, written information provided, and written informed consent obtained from those willing to participate in the study. Following surgery, all consented patients were re-screened on the ward prior to their first potential VR session. Patients with post-surgery delirium, cognitive impairments, severe hearing or vision issues, incompatible eyeglasses, non-English speakers, dementia, epilepsy, other neurological conditions, post-surgery complications (e.g. ventilation or oxygen therapy), sensitivity to motion or light, inability to complete assessments, or involvement in other studies were excluded at this stage. Only patients who continued to meet the inclusion criteria and were clinically stable were offered VR. The recruitment period was from 2nd August 2023 to 4th December 2023. Staff, including nurses and volunteers working on the four surgical wards, were invited to participate, as we wished to understand their views and potential involvement in VR delivery as future providers. All permanent and bank nursing staff and registered volunteers on these wards were eligible, regardless of previous VR experience, provided they were able to give informed consent and were involved in routine post-operative care. Prior use or familiarity with the DR.VR system was not required. However, despite initial informal interest, no staff or volunteers were ultimately recruited during the study period, and acceptability data were, therefore, obtained only from patients.

### Sampling method

Based on an estimated 10 eligible patients per month, the study aimed to consent 20 participants over a 4-month period. As a feasibility study, no formal power calculation was performed, and hypothesis testing was not the primary focus. However, to aid interpretation, we report precision estimates for key continuous variables (means with standard deviations, as appropriate).

### Intervention

**Fig. 1:**
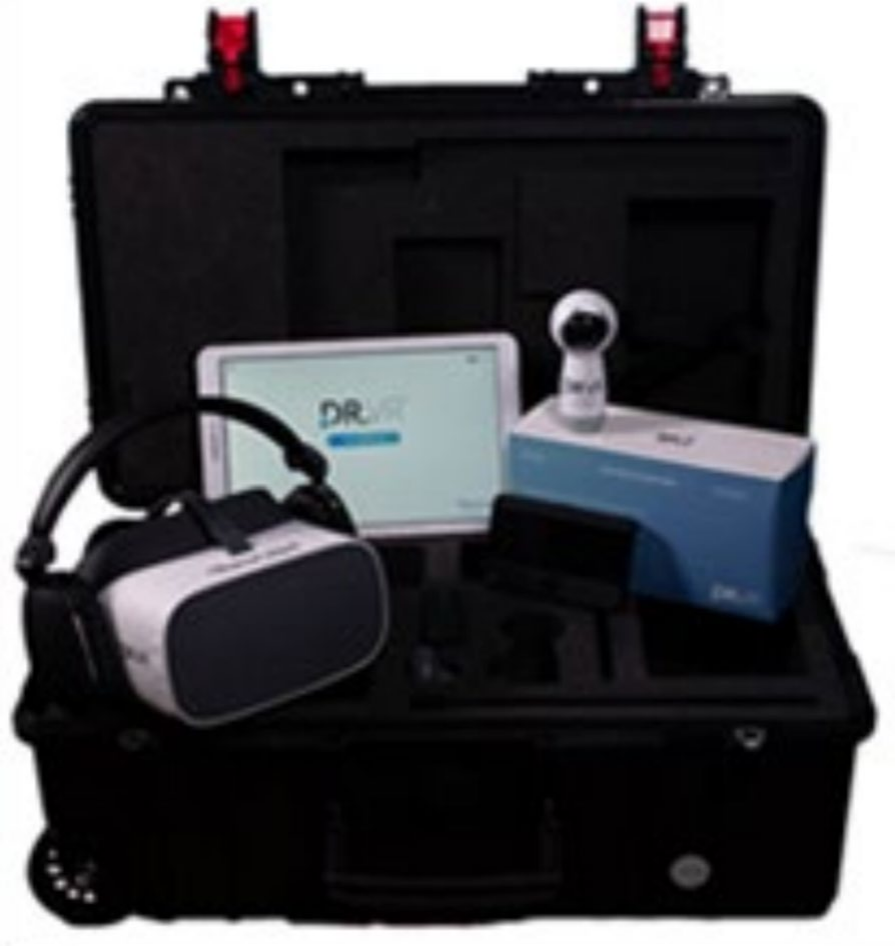
Virtual reality equipment used

**Fig. 2:**
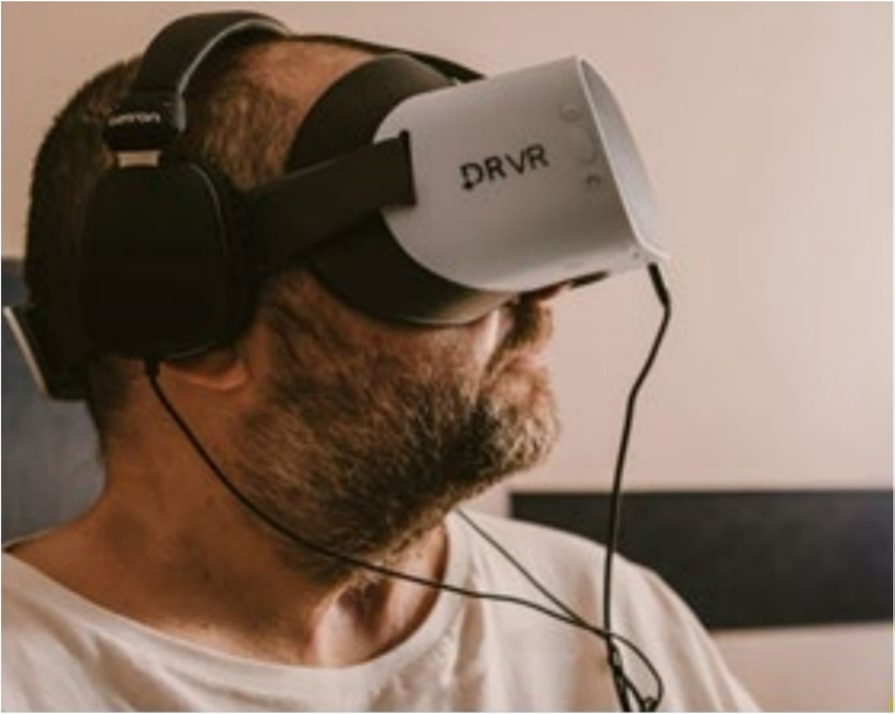
Person using a virtual reality headset

Beginning 24 h post-operatively, all participants were offered up to three VR sessions during their inpatient stay, up to the point of hospital discharge. Sessions were scheduled on separate occasions (e.g. on consecutive or alternate days), depending on clinical stability, participant availability, and length of stay. Each session was planned to last approximately 7.5 min and all participants undertook the total duration of the sessions. The VR system used was DR.VR (Rescape Innovation, Cardiff, UK), which provides a range of immersive experiences (Figs. 1 and 2; Additional file 2). All infection control protocols were adhered to during the study. DR.VR is a standalone head-mounted display with integrated headphones, supplied and maintained by Rescape Innovation under an existing service agreement with the Trust. No handheld controllers were used. Participants interacted with the system minimally. The first author selected and started the chosen module, and participants experienced the content as an immersive audio-visual environment from their bed or chair. The experiences therefore functioned primarily as guided, passive modules (e.g. nature scenes, travel environments, relaxation and mindfulness exercises, and light gameplay) rather than highly interactive games.

Participants could choose from a menu of experiences, including ocean and underwater scenes, countryside and city exploration, and simulated activities such as surfing or skydiving, which they viewed from their bed or chair. Each module included accompanying audio (e.g. ambient nature sounds, music, or a calm narrated voiceover). Experiences were non-interactive: participants did not use hand controllers, and their role was to attend to the immersive audio-visual content. For each session, participants were invited to select either relaxation-focused content (e.g. mindfulness, meditation, breathing exercises) or more stimulating, game-based modules, supported by the first author to ensure safety and comfort. The first author is a nurse with over 9 years of clinical experience, received training in the use of VR through an online session led by the software’s commercial representative. This representative holds an existing agreement with the Trust to maintain the device and provide user training in clinical areas. The session covered the safe operation of the DR.VR system and how to identify signs of wooziness, nausea, or sensitivity to motion. The training session was recorded for reference and used for ongoing support throughout the study period. VR sessions were delivered by the first author as part of her internal research internship, her initial steps into research, under the supervision of the second author, a senior clinical academic with more than 20 years of experience in both quantitative and qualitative research.

All patients received standard post-operative multimodal analgesia as per hospital protocol, typically comprising regular paracetamol and non-steroidal anti-inflammatory drugs (where not contraindicated) with opioid medication prescribed as required.

### Outcome measures

A screening log recorded patient eligibility, consent, and retention, along with demographic data such as age and gender on the DR. VR system. We collected only essential demographic data (age and gender) to minimise participant burden and focus on the acceptability and usability of the VR intervention. We did not collect detailed data on the dose or timing of analgesic or anxiolytic medications during the study. No personal identifiers were stored and participants were assigned a unique anonymised study number. We had planned to obtain feedback from nursing staff and volunteers regarding the acceptability and practicality of supporting patients to use DR.VR. However, due to workload pressures and competing priorities on the wards, no staff or volunteers were recruited during the study period, and staff-focused acceptability data were therefore not collected.

Aiming to recruit 20 participants, retention rate was calculated by comparing the number of participants recruited and the number of participants that completed at least one session of the VR experience. This number was also compared to the number of participants who signed consent and declined to complete at least one session of the VR experience or withdrew from the study.

**Fig. 3:**
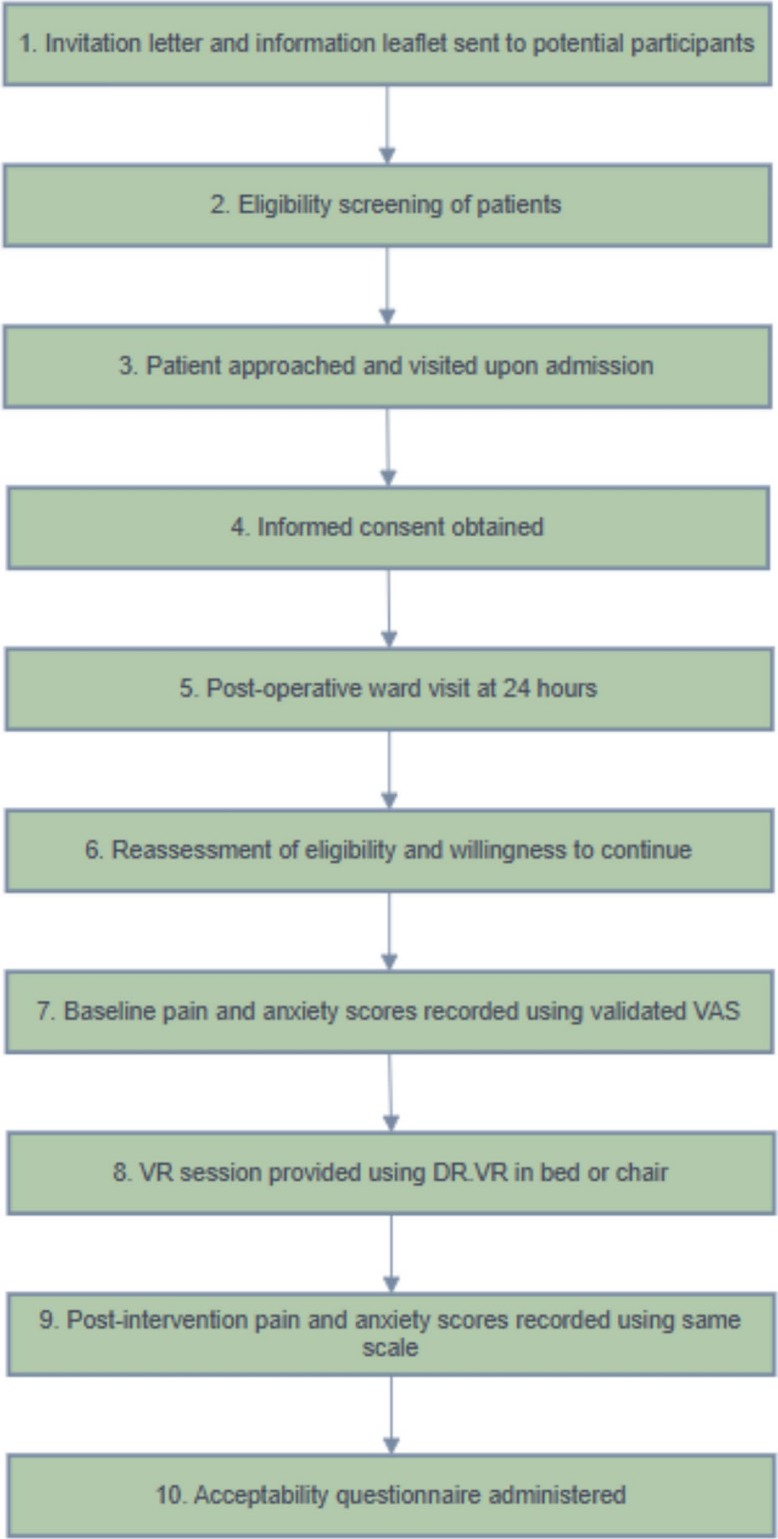
An overview of the study procedure

Pain and anxiety levels were measured immediately before and immediately after each VR session using a validated visual analogue rating scale (0, no pain to 10, maximal pain) and a numeric rating scale (0, no anxiety to 10, maximal anxiety), electronically by VR tablet and participants entered their responses directly [32–34] (Additional file 3). Within 10 min of participants-VR sessions, participants completed an anonymous paper-based questionnaire using a five-point Likert scale to assess VR acceptability. This included space for free text comments for additional feedback. All questionnaires were anonymous and completed by the participants themselves (Additional file 4) An overview of the study procedure is presented in Fig. 3.

Acceptability of the VR intervention was assessed using a custom not validated questionnaire developed by the study team in collaboration with patients. The questionnaire included items on overall experience, perceived benefits, and ease of use of the VR equipment. It was reviewed by four patients and four staff members, with input from a patient representative, to ensure clarity, relevance, and ease of understanding. While not a previously validated instrument, this process ensured that the questionnaire was appropriate and meaningful for the study population.

### Data analysis

All quantitative data (screening log, demographic information, pain and anxiety scores, and questionnaire responses) were entered into Microsoft Excel by the first author. Data entry was checked for accuracy by the third author. No missing data were observed in this study. Analyses were based on available data for each variable. Discrete variables were expressed as absolute frequencies (n/[%]). Continuous variables (e.g. age) were summarised using means and standard deviations. Qualitative free-text feedback from the acceptability questionnaires was analysed using conventional content analysis. The first author conducted initial open coding, grouping similar statements into categories. The third author, an experienced qualitative researcher with more than 20 years of expertise in content analysis, independently reviewed the coding framework and a sample of the coded data. Discrepancies were discussed and resolved by consensus, and the final themes were agreed by both authors to enhance trustworthiness of the findings.

### Ethical approval

UK ethical approval was obtained from HRA and Health and Care Research Wales (HCRW), UK reference number 21/NW/0359. All participants were informed and gave consent to participate.

## Results

### Participants

Of the 23 participants who consented, 10 were male (43.48%) and 13 were female (56.52%). The mean age of patients, calculated using age-range midpoints, was 69.23, ± 11.64 years. The mean age of patients who declined participation was 65.67 years, compared with 68.96 years among those who consented.

### Primary outcome

**Fig. 4:**
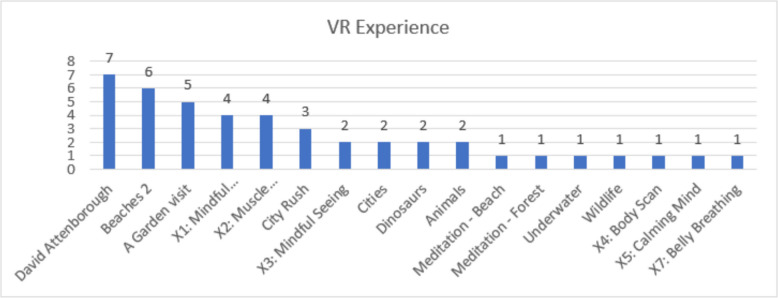
Frequency of VR sessions completed

Of the 145 patients were scheduled for TKR or RTKR surgery, 57 were identified and approached by clinicians. 42 (73.7%) agreed to receive the Patient Information Sheet (PIS). 11 (26.19%) of those were excluded for not meeting the inclusion criteria: five (11.9%) due to language barriers; four (9.52%) due to neurological diagnoses; and two (4.76%) due to significant visual and/or hearing impairments. Eight patients (19.04%) declined to participate. Of these 23 participants consented (54.76%), three (13.04%) not complete any VR sessions due to due to nausea and vomiting before the VR experience, a spasm or declining without reason. The remanding 20 participants completed at least one VR session, reflecting an 86.96% retention rate. In total, 44 VR sessions were completed, with a mean of 2.2 ± 0.6 sessions per participant. Twelve participants undertook TKR and six RTKR. Two participants completed 1 session, twelve participants completed 2 sessions, and five participants completed 3 sessions. The most completed sessions were *David Attenborough*, with six participants, followed by *A Garden Visit* with five participants, *and Beaches 2* with four participants, while all other sessions attracted three or less participants. Figure 4 show the frequency of VR sessions completed.

### Secondary outcomes

**Fig. 5:**
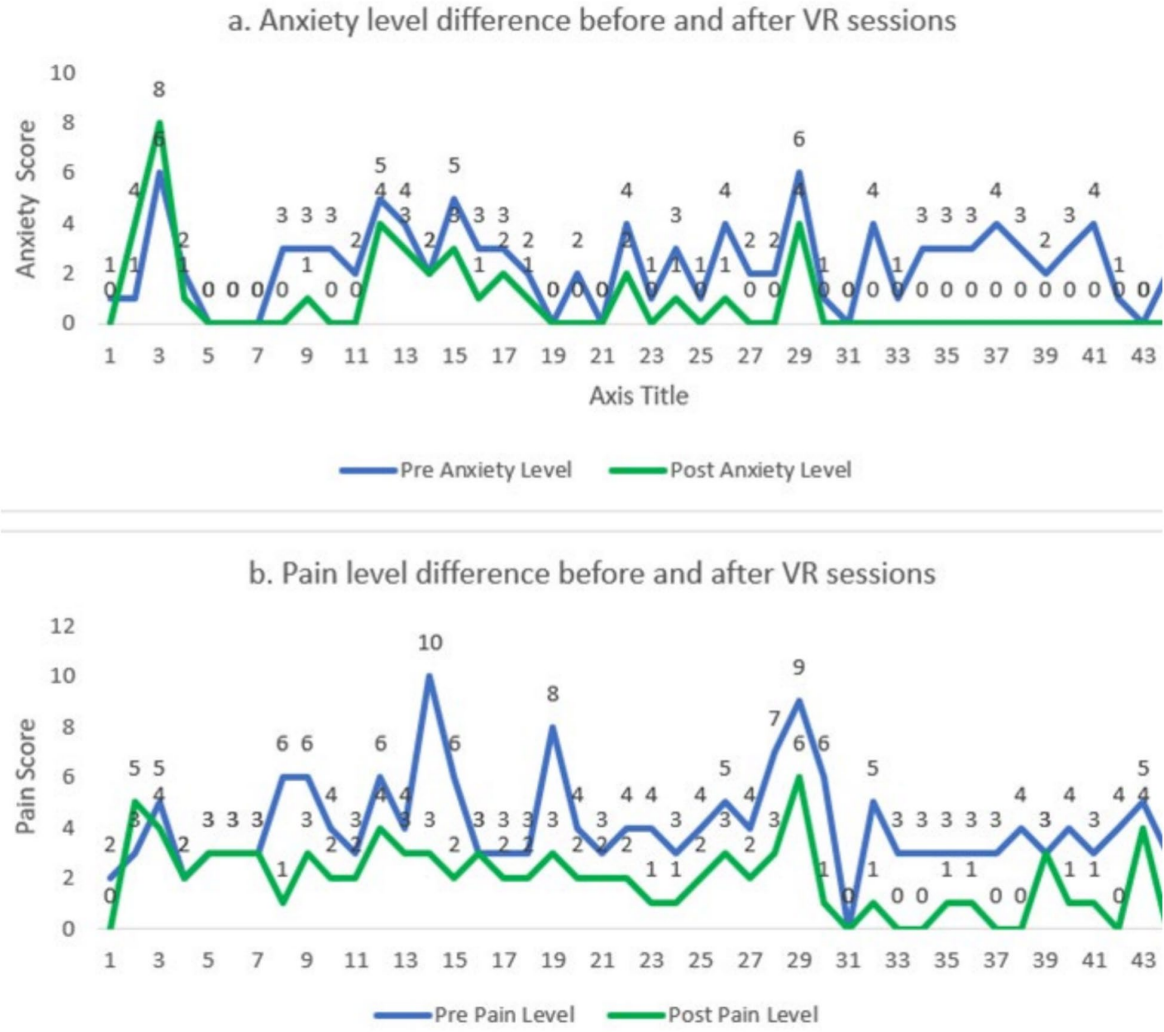
Anxiety and pain level before and after sessions

Forty-three anxiety and pain scores were recorded before and after each session. Figure 5 shows the differences in anxiety and pain levels before and after the sessions.

#### Pain

The mean pre-session pain score was 4.14 ± 1.88, which decreased to 1.98 ± 1.44 following VR exposure. Across all sessions: 81.4% of participants reported a reduction in pain; 16.3% reported no change, and 2.3% reported an increase.

#### Anxiety

The mean pre-session anxiety score was 2.36 ± 1.63, decreasing to 0.86 ± 1.64 post-session. Overall: 77.3% reported a reduction in anxiety; 18.2% reported no change, and 4.5% reported an increase.

#### Acceptability

All 20 participants provided feedback on their VR experience. Overall, the intervention was well-received. Table 1 shows full results. The majority enjoyed the experience (95% agreement) and found the information clear (100% agreement). Most participants reported feeling distracted from post-surgical pain (85%) and experienced a sense of well-being (85%). Anxiety reduction was reported by 85% of participants, while physical comfort showed more mixed responses. Participants strongly endorsed the intervention, with 90% indicating they would recommend VR to friends or family. The VR equipment was generally rated as easy to use, and no adverse effects such as fatigue, headache, or dizziness were reported.

**Table 1:** Participant feedback on VR experience (*n* = 20)

Statement	Strongly Agree	Agree	Undecided	Disagree	Strongly Disagree
I enjoyed my experience	12 (60%)	7 (35%)	1 (5%)	0	0
The information provided was clear	14 (70%)	6 (30%)	0	0	0
I felt distracted from my post-surgical pain	11 (55%)	6 (30%)	3 (15%)	0	0
Using VR gave me a sense of well-being	11 (55%)	6 (30%)	2 (10%)	1 (5%)	0
I was physically pain-free during the experience	4 (20%)	2 (10%)	3 (15%)	9 (45%)	2 (10%)
My anxiety level decreased during the experience	10 (50%)	7 (35%)	2 (10%)	0	1 (5%)
I would recommend VR to friends/family	13 (65%)	5 (25%)	0	2 (10%)	0
Interaction devices were easy to use	13 (65%)	4 (20%)	2 (10%)	1 (5%)	0
Experienced fatigue, headache, nausea, dizziness	0	0	0	0	0

Qualitative feedback highlighted the immersive and calming nature of the VR sessions. Participants suggested longer session durations, improved content quality, and quieter settings to enhance the experience. Comments included “Longer views would enhance the whole experience” (P011); “Make it clearly for small details” (P017); and “Being in a quieter environment” (P007). These findings indicate that VR is a highly acceptable and engaging intervention for post-surgical patients, with potential for further refinement.

## Discussion

The primary aim of this study was to evaluate the feasibility and acceptability of using VR as a non-pharmacological intervention for managing pain and anxiety in patients undergoing TKR and RTKR. Findings provide preliminary evidence supporting VR’s potential benefits in post-operative care. Recruitment and retention rates were promising, that aligns with previous feasibility studies highlighting the potential of VR in healthcare settings. These preliminary findings suggest that VR is an accepted tool for managing both pain and anxiety post TKR and RTKR in clinical settings [3, 35]. VR’s potential to relieve pain is likely multifactorial. Distraction from nociceptive stimuli through immersive, engaging environments is one proposed mechanism, alongside possible effects on relaxation, mood, and anxiety [36]. These findings are consistent with prior research demonstrating VR’s ability to modulate pain perception through immersive distraction techniques [36, 37]. VR likely diverts attention away from nociceptive stimuli by engaging the brain’s sensory and cognitive pathways in a novel and stimulating environment [18, 38, 39]. Additionally, systematic reviews have highlighted VR’s role in reducing procedural pain and anxiety across diverse populations, including those undergoing burn wound care [18, 19, 40] endoscopies [41–45] and colonoscopies [46–48]. Moreover, anxiety levels reducing during VR sessions aligning with findings from other studies and suggests that VR promotes relaxation and lowers anxiety, especially in patients undergoing stressful treatments [47]. VR has been shown to significantly reduce pre-treatment anxiety in chemotherapy [47, 48] patients and improve psychological well-being through relaxation techniques like mindful breathing. Its immersive nature helps focus on positive stimuli, counteracting anxiety-related negative emotions [49, 50]. Participants’ feedback was positive, with participants noting that the VR sessions effectively distracted them from their pain and reduced anxiety. This echoes findings [19, 51] that demonstrated VR’s efficacy in managing pain in hospitalised patients. Additionally, no adverse events, such as nausea or dizziness, were reported during the study, reinforcing VR’s safety profile. This is consistent with findings [52–56] that show minimal adverse effects in their meta-analysis of VR interventions for post-operative pain. Our findings contribute to a future study, emphasising the utility of VR in post-surgical settings. It is important to note that this study was not designed to robustly assess reductions in pain or anxiety, and only immediate post-session scores were collected. Therefore, conclusions about VR’s efficacy in reducing pain and anxiety should be interpreted with caution, as these findings reflect very short-term effects rather than sustained benefits. Furthermore, given the limited number of confounders assessed, the results cannot confirm causality. Moreover, the study encountered challenges in recruiting ward staff and volunteers to assist with VR implementation. Despite initial interest from staff, none participated, indicating potential barriers such as time constraints or insufficient training. Future studies should address these barriers through tailored staff training programmes and robust stakeholder engagement [57, 58]. These suggestions highlight that, in addition to the delivery platform, the specific content and its duration are critical to perceived effectiveness. Improving visual fidelity, expanding the range of environments, and offering longer or more flexible sessions may enhance immersion and perceived benefit. Future iterations of the intervention should therefore prioritise co-design of VR content with patients and clinicians to optimise engagement and therapeutic value.

The study’s strengths lie in its innovative approach and adherence to rigorous ethical and methodological standards, including compliance with the TREND checklist. However, several limitations warrant consideration. While this feasibility study limited demographic data collection to reduce participant burden, future larger-scale studies should incorporate additional variables such as ethnicity, socioeconomic status, education level, and comorbidities to assess representativeness and explore potential subgroup differences. Including measures such as the Index of Multiple Deprivation and broader representation of ethnic minorities will enhance the validity and generalisability of results. This will support a more comprehensive evaluation of the VR intervention’s effectiveness across diverse patient populations. Moreover, as a single-centre design and small sample size limit the generalisability. Additionally, most participants experienced only one VR session due to early discharge, preventing an assessment of VR’s long-term efficacy. Similar challenges were reported in other feasibility studies, underscoring the need for multi-centre trials with larger samples [3, 59, 60]. Given that two participants completed only a single VR session while still reporting perceived benefits, future research should explore whether a single, longer session may be more practical and potentially as effective, or even more effective, than multiple shorter sessions in this cohort. Additionally, reasons for completing only a single session, such as early discharge, represent data that should be systematically collected in future studies. Importantly, our findings reflect the specific DR.VR modules deployed (e.g. calming natural scenes and relaxation-focused content) rather than VR as a homogeneous intervention, and future trials should compare different content types (e.g. mindfulness vs interactive games) to determine which are most beneficial for patients following joint replacement.

Although all participants followed the hospital’s standard post-operative pain protocol, we did not collect detailed data on analgesic or anxiolytic dosing or timing. Future studies should collect comprehensive medication data to reduce risk of bias and better understand potential confounding factors. Encouragingly, the study exceeded its recruitment target, and participant willingness suggests strong potential for scaling. These findings support the design of a future definitive trial, ideally a multicentre, randomised study with a larger and more diverse sample. Furthermore, future trial should also refine inclusion criteria, improving participant engagement, especially in coordination with nurses on the ward and volunteers, and improving the intervention itself. This could include longer or repeated VR sessions, including post-discharge use at home. Finally, in agreement with the NICE guidelines 2021 [61], promoting person-centred, non-pharmacological approaches to pain management is essential. Integrating VR into routine post-operative care, including care after discharge will be critical steps towards effective implementation.

## Conclusion

In conclusion, for our knowledge, this is the first feasibility quasi- experimental study which provides preliminary evidence supporting the use of VR as a safe, acceptable, and potentially effective adjunct for managing pain and anxiety in patients undergoing TKR and RTKR. These findings lay the groundwork for future definitive trials and highlight VR’s potential as a transformative tool in post-operative care. Further research is needed to optimise intervention protocols, evaluate long-term outcomes, and explore VR’s broader applications in healthcare, which will be our future study.

## Supplementary Information

Additional file 1.

Additional file 2.

Additional file 3.

Additional file 4.

## Data Availability

The datasets used and/or analysed during the current study are available from the corresponding author on reasonable request.

## Abbreviations

DR.VR: Digital Reality Virtual Reality (VR system by Rescape Innovation)
HCRW: Health and Care Research Wales
HRA: Health Research Authority
MRC: Medical Research Council
NHS: National Health Service
NICE: National Institute for Health and Care Excellence
PIS: Patient Information Sheet
RTKR: Revision Total Knee Replacement
TKR: Total Knee Replacement
TREND: Transparent Reporting of Evaluations with Nonrandomised Designs
UK: United Kingdom
VR: Virtual Reality
