# Supplementary material for: A feasibility and acceptability of virtual reality as a pain relief measure post primary and revision total knee replacement surgery in a hospital setting: quasi-experimental study
Source: BMC Musculoskelet Disord. 2026 Mar 10;27:323. doi: 10.1186/s12891-026-09599-y (PMC13085278; doi:10.1186/s12891-026-09599-y)
Supplement: Supplementary file 2 — Additional file 2. [file 12891_2026_9599_MOESM2_ESM.docx]

**DR VR Content Information**

**RELAX**

| NAME | DESCRIPTION | DURATION |
| --- | --- | --- |
| Animals | "In the Prescence of Animals" Feel the presence of massive bison, a million monarch butterflies and the endangered jaguar. Narrated by Dominic West. With thanks to C1 VR for supplying this experience. | 00:04:30 |
| Beaches | Enjoy relaxing while visiting 7 stunning secret beaches. This mindfulness experience is designed to reduce stress and help you relax. | 00:07:30 |
| Beaches 2 | Enjoy relaxing while visiting stunning beaches. This mindfulness experience is designed to reduce stress and help you relax. | 00:07:30 |
| Dinosaurs | A rare trip back in time to our very special Dino Safari Park. You'll encounter some of the biggest and most ferocious creatures that have ever roamed the Earth. | 00:09:47 |
| David Attenborough | Take a mindful trip through an enchanting forest guided by the calming voice of Sir David Attenborough. Arrive at a beautiful waterfall and throw pebbles into the cool deep lake. | 00:06:39 |
| Gardens | A wonderful opportunity to visit two magical gardens in early summer and late autumn. With thanks to National Gardens Scheme and Hospice UK. | 00:07:35 |
| Mindful Breathing | This starter session will teach you to be mindful about your breathing, allowing you to check in on your body and learn to control your breathing and how this can affect any anxiety or stress. | 00:07:31 |
| Muscle Relaxation | This session will teach you a simple technique to relax your muscles. Remember any tasks practiced in VR can be used day to day. Close your eyes and you can be back on the beach. | 00:07:00 |
| Mindful Seeing | This session will teach you how to mindfully look at the world. To stop and be in the moment, to take time to look at something beyond its label, and look at its colour, texture and shape. | 00:07:31 |
| Body Scan | This session is a wonderful mindfulness session to reduce anxiety and stress. It will allow you to accept, with gentle curiosity, how your body is feeling. | 00:07:31 |
| Calming Mind | This session connects you with the water in a lake. Watching the ripples of water as you gain the space to calm your mind and bring perspective to your thoughts. | 00:07:31 |
| Mindful Listening | This session gives us the space to really listen to the world around us. To stop and be within the moment. Mindful listening will allow you to take this practice into everyday life. really listening to the world around you | 00:07:31 |
| Belly Breathing | This session will help you practice a more full and deep pattern of breathing called belly breathing. This may take some time to perfect and can be difficult at the start, but once learnt it is a technique you can use in everyday life. | 00:07:31 |
| Cities | Travel to various cities from around the world. Enjoy your time there whilst listening to the history behind some of the world's most famous landmarks. | 00:07:29 |
| Country Stroll | A relaxation experience while you stroll through the beautiful countryside and mountain ranges. Listen with a choice of poem readings. | 00:07:35 |
| Relaxation | Give yourself some time to relax and escape with this relaxation experience while sitting and visiting some beautiful environments around the world. | 00:07:30 |
| Relaxation 2 | Give yourself some time and relax with this mindfulness experience while sitting in some beautiful environments. | 00:07:30 |
| Sleep Relaxation | This guided relaxation experience will help you to relax after a long day, and take a moment to re-center yourself. Choose from a variety of exercises from Muscle Relaxation to Belly Breathing from the commentary panel on the right, and enjoy watching the sunset in a beautiful savanna. | 00:07:29 |
| Wild Hikes | A wild hike off the beaten track to amazing landscapes, remote beaches, extraordinary mountain ranges and lush green forests. | 00:07:30 |
| Wildlife | Sit and experience life up close and personal with some of the worlds most endangered animals from being as small as a bug to looking up at the tallest giraffe! | 00:07:30 |
| Space | Who hasn't dreamt of flying across the universe in your own spaceship? Now you can! \| Visit the surface of mars and the rings of Saturn, with a few surprise stops along the way. | 00:10:12 |
| Travel | The world is full of wonder and beauty. You'll get to travel to see some of the hidden wonders of the world, from exploding volcanoes to salmon fishing with black bears. | 00:07:30 |
| Underwater | From the warm Caribbean Sea to the clear waters of the Pacific Ocean, you'll get to swim with some of the most majestic and endangered creatures on our planet. | 00:07:33 |
| Meditation Beach | Relax and practice your breathing in relaxing environments. | N/A |
| Meditation Winter | Relax and practice your breathing in relaxing environments | N/A |
| Meditation Forest | Relax and practice breathing in relaxing environments | N/A |

**PLAY/GAMES**

| **NAME** | **DESCRIPTION** | **DURATION** |
| --- | --- | --- |
| Arctic Beats | A relaxing musical adventure. Collect the golden rings for points and the purple rings for extra time. | N/A |
| City Rush | Fly through towers, avoid obstacles, score the highest! | N/A |
