# Supplementary figures and images for: A feasibility and acceptability of virtual reality as a pain relief measure post primary and revision total knee replacement surgery in a hospital setting: quasi-experimental study
Source: BMC Musculoskelet Disord. 2026 Mar 10;27:323. doi: 10.1186/s12891-026-09599-y (PMC13085278; doi:10.1186/s12891-026-09599-y)

Virtual Reality Anxiety level score


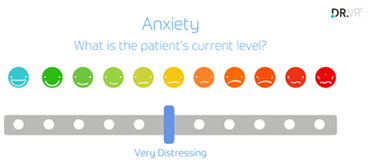


Virtual Reality pain level score


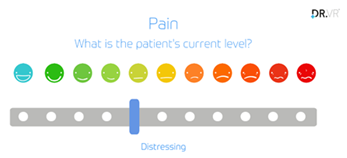

Supplement: Supplementary file 3 — Additional file 3. [file 12891_2026_9599_MOESM3_ESM.docx]
