# Supplementary material for: A feasibility and acceptability of virtual reality as a pain relief measure post primary and revision total knee replacement surgery in a hospital setting: quasi-experimental study
Source: BMC Musculoskelet Disord. 2026 Mar 10;27:323. doi: 10.1186/s12891-026-09599-y (PMC13085278; doi:10.1186/s12891-026-09599-y)
Supplement: Supplementary file 4 — Additional file 4. [file 12891_2026_9599_MOESM4_ESM.docx]

**Questionnaire to understand patient’s view on the use of virtual reality and pain control post-surgery**

This questionnaire aims to understand patient’s views on use of virtual reality and pain control post-surgery.

Your voluntary participation should take approximately 10 minutes. You have the right to withdraw your consent or discontinue participation at any time without given any justification.

Your participation in this study involves no major risks whatsoever, be it physical or emotional, it may be of value for patient future pain treatments.

Any information you give to us will be kept confidential and anonymised in all published and written reports resulting from the study. The questionnaire is strictly anonymous.

Please circle the number that best scores your opinion between 1 = ‘I don’t agree at all’ to 5 = ‘I totally agree’ on virtual reality questions in the next page

| Strongly disagree | Disagree | Undecided | Agree | Strongly agree |
| --- | --- | --- | --- | --- |
| 1 | 2 | 3 | 4 | 5 |

|  | **Strongly disagree** | **Disagree** | **Undecided** | **Agree** | **Strongly Agree** |
| --- | --- | --- | --- | --- | --- |
| 1. I enjoyed my experience | 1 | 2 | 3 | 4 | 5 |
| 1. The information provided was clear. | 1 | 2 | 3 | 4 | 5 |
| 1. I felt distracted from my post-surgical pain | 1 | 2 | 3 | 4 | 5 |
| 1. Using the virtual reality gave me a sense of well-being. | 1 | 2 | 3 | 4 | 5 |
| 1. I was physically pain free during the experience. | 1 | 2 | 3 | 4 | 5 |
| 1. My anxiety level decreased during the experience. | 1 | 2 | 3 | 4 | 5 |
| 1. I would recommend the use of virtual reality to friends and family for post-surgical use. | 1 | 2 | 3 | 4 | 5 |
| 1. I found the interaction devices (Googles, headset and keyboard) easy to use. | 1 | 2 | 3 | 4 | 5 |
| 1. I felt fatigued/tired during my interaction | 1 | 2 | 3 | 4 | 5 |
| 1. I experienced a headache during the interaction | 1 | 2 | 3 | 4 | 5 |
| 1. I experienced eyestrain | 1 | 2 | 3 | 4 | 5 |
| 1. I felt nauseous | 1 | 2 | 3 | 4 | 5 |
| 1. I experienced a feeling of “fullness of the head” during my interaction | 1 | 2 | 3 | 4 | 5 |
| 1. I felt dizzy/lightheaded during the interaction | 1 | 2 | 3 | 4 | 5 |
| 1. I experienced vertigo (environment moving or spinning) during my interaction. | 1 | 2 | 3 | 4 | 5 |

Thank you for completing the questionnaire

Research contact: Queen Adeyanju

queen.adeyanju@nhs.net

Do you have suggestions to improve this virtual reality environment?

17. What were the negative points about your experience?

16. What were the positive points about your experience?
